# Relationship between Acute Phase of Chronic Periodontitis and Meteorological Factors in the Maintenance Phase of Periodontal Treatment: A Pilot Study

**DOI:** 10.3390/ijerph120809119

**Published:** 2015-08-05

**Authors:** Noriko Takeuchi, Daisuke Ekuni, Takaaki Tomofuji, Manabu Morita

**Affiliations:** 1 Departments of Preventive Dentistry, Okayama University Graduate School of Medicine, Dentistry and Pharmaceutical Sciences, 2-5-1 Shikata-cho, Kita-ku, Okayama 700-8558, Japan; E-Mails: dekuni7@md.okayama-u.ac.jp (D.E.); tomofu@md.okayama-u.ac.jp (T.T.); mmorita@md.okayama-u.ac.jp (M.M.); 2 Advanced Research Center for Oral and Craniofacial Sciences, Okayama University Dental School, 2-5-1 Shikata-cho, Kita-ku, Okayama 700-8558, Japan

**Keywords:** acute phase, chronic periodontitis, barometric pressure, supportive periodontal therapy, autoregressive integrated moving average model

## Abstract

The acute phase of chronic periodontitis may occur even in patients during supportive periodontal therapy. However, the details are not fully understood. Since the natural environment, including meteorology affects human health, we hypothesized that weather conditions may affect occurrence of acute phase of chronic periodontitis. The aim of this study was to investigate the relationship between weather conditions and acute phase of chronic periodontitis in patients under supportive periodontal therapy. Patients who were diagnosed with acute phase of chronic periodontitis under supportive periodontal therapy during 2011–2013 were selected for this study. We performed oral examinations and collected questionnaires and meteorological data. Of 369 patients who experienced acute phase of chronic periodontitis, 153 had acute phase of chronic periodontitis without direct-triggered episodes. When using the autoregressive integrated moving average model of time-series analysis, the independent covariant of maximum hourly range of barometric pressure, maximum hourly range of temperature, and maximum daily wind speed were significantly associated with occurrence of acute phase of chronic periodontitis (*p* < 0.05), and 3.1% of the variations in these occurrence over the study period were explained by these factors. Meteorological variables may predict occurrence of acute phase of chronic periodontitis.

## 1. Introduction

Periodontitis is one of the most widespread chronic diseases and is characterized by gingival bleeding, periodontal pocket formation, destruction of connective tissue attachment, and alveolar bone resorption. Based on a Japanese national survey, 42.5% of Japanese exhibited periodontitis [1]. The primary etiological agent for periodontitis is dental plaque bacteria [2,3]. The majority of periodontal tissue destruction is caused by abnormal host responses to these microorganisms and their products [4,5].

For the long-term stability of successfully treated periodontitis, supportive periodontal therapy (periodontal maintenance) is needed and is regarded as an integral part of overall periodontal management [6,7,8]. Although dentists have been making every effort to maintain periodontal health and prevent tooth loss during supportive periodontal therapy, acute phase of chronic periodontitis may occur in patients and has been regarded as one of the possible complications of supportive periodontal therapy [9]. A review [10] suggested that there were intraoral risk factors related to acute phase of chronic periodontitis. However, clinicians sometimes encounter patients suffering from periodontal abscess without any intraoral factors. Therefore, there may be a relationship between acute phase of chronic periodontitis and extraoral factors.

The natural environment including meteorology affects human health. Weather conditions have been found to be related to blood pressure [11,12,13], myocardial infarction [14,15], heatstroke [16], stroke [17], paediatric trauma [18], mortality [19], asthma [20,21], depression [22,23], rheumatoid arthritis [24,25], and pain in general [26]. In dentistry, some recent research suggests that meteorology is related to temporomandibular disorders [27] and oral pain [28].

Based on these findings, we hypothesized that weather conditions may cause occurrence of acute phase of chronic periodontitis. The aim of this study was to investigate the relationship between weather conditions and occurrence of acute phase of chronic periodontitis in patients under supportive periodontal therapy. Since meteorological variables influence one another [29], we chose temperature, rainfall, barometric pressure, wind speed, hours of sunlight, and humidity as meteorological variables.

## 2. Methods

### 2.1. Study Population

This study was performed at the Preventive Dentistry clinic in Okayama University Hospital in Japan from November 2011 to November 2013. In the clinic, all patients received supportive periodontal therapy that included non-surgical periodontal therapy consisting of oral examination, oral hygiene instructions, supra/sub-gingival debridement and scaling, and root-planing every 3–4 months [30]. The patients who were diagnosed with acute phase of chronic periodontitis under supportive periodontal therapy were selected for this study. Criteria for the acute phase of chronic periodontitis included at least two of the following clinical signs or symptoms; pain, swelling, redness or a feeling of warmth in the periodontal lesion [31,32]. Exclusion criteria were as follows: patients who had cancer, hospitalized patients, and patients with missing data. The patients who lived outside of Okayama’s local meteorological area were also excluded. This study was approved by the Ethics Committee of Okayama University Hospital (No. 502, 692). After obtaining written informed consent, the dentists completed a detailed medical questionnaire and oral examination as follows.

### 2.2. Oral Examination

All clinical procedures were performed by four trained and calibrated dentists (T.T., M.M., D.E., and N.T.). To distinguish other diseases including apical periodontitis, tooth fracture and trauma, acute phase of chronic periodontitis were confirmed by X-ray and or oral examination.

### 2.3. Questionnaires

The dentists took the history of acute phase of chronic periodontitis. They collected information about the date, place, and possible trigger that the patients were aware of, and any acute symptoms, such as physical stress, mental stress, occlusal trauma, insufficiency for oral hygiene, and so on. When the patients could not identify the trigger clearly and dentists could not determine the clinical factors affecting acute phase of chronic periodontitis, such cases were defined as acute phase of chronic periodontitis without direct-triggered episodes.

### 2.4. Meteorological Measurements

Meteorological data were acquired from the local meteorological office at Okayama City. The data included the following: (1) mean daily wind speed (m/s), (2) maximum daily wind speed (m/s), (3) minimum daily wind speed (m/s), (4) mean daily barometric pressure (hPa), (5) maximum daily barometric pressure (hPa), (6) minimum daily barometric pressure (hPa), (7) daily range of barometric pressure (hPa), (8) maximum hourly increase in barometric pressure (hPa), (9) maximum hourly decrease in barometric pressure (hPa), (10) total hours of sunlight, (11) total daily rainfall (mm), (12) mean daily temperature (°C), (13) maximum daily temperature (°C), (14) minimum daily temperature (°C), (15) daily range of temperature (°C), (16) maximum hourly increase in temperature (°C), (17) maximum hourly decrease in temperature (°C), (18) mean daily humidity (%), (19) maximum daily humidity (%), (20) minimum daily humidity (%), and (21) daily humidity range [29].

### 2.5. Statistical Analysis

A multivariate autoregressive integrated moving average model (ARIMA) was performed to identify whether meteorological variables might predict occurrence of acute phase of chronic periodontitis using Box-Jenkins methodology [29,33]. Models were identified by determining ARIMA model orders (p, d, q) using autocorrelation and partial autocorrelation. The model parameters were estimated by the unconditional least squares method. Finally, the adequacy of the model was checked and statistical significance of the parameters determined [33]. After identifying the multivariate transfer function models, the cross-correlation function was determined by estimating the correlations between occurrence of acute phase of chronic periodontitis at different time lags and metrological valuables. The final model was determined by the highest biological plausibility and determination coefficient (r2). All data were analyzed using the Statistical Package for the Social Sciences (21.0J for Windows; SPSS Japan, Tokyo, Japan). Values of *p* < 0.05 were considered to represent statistically significant differences.

## 3. Results

Table 1 presents the characteristics of the study population. Of a total of 20,034 patients who had received supportive periodontal therapy at the hospital clinic, 369 patients developed acute phase of chronic periodontitis (1.84% of all patients). The number of acute phase of chronic periodontitis without direct-triggered episodes was 153 (0.76%) and the mean age of patients was 68.7 years (SD = 11.2). The proportion of females was 73.9%.

**Table 1:** Characteristics of the study population.

Age	Male	Female	Total
**Patients Who Had Received Supportive Periodontal Therapy**			
20–29	114	138	252
30–39	135	444	579
40–49	434	922	1356
50–59	726	2235	2961
60–69	1962	4766	6728
70–79	1842	4031	5873
80–	942	1343	2285
Total	6155	13,879	20,034
**Patients with Acute Phase of Chronic Periodontitis**			
20–29	0	0	0
30–39	1	4	5
40–49	3	9	12
50–59	9	25	34
60–69	33	114	147
70–79	34	89	123
80–	13	35	48
Total	93	276	369
**Patients with Acute Phase of Chronic Periodontitis without Direct-Triggered Episodes**			
20–29	0	0	0
30–39	0	2	2
40–49	1	4	5
50–59	6	7	13
60–69	10	48	58
70–79	18	40	58
80–	5	12	17
Total	40	113	153

The mean probing pocket depth at teeth with occurrence of acute phase of chronic periodontitis was 5.1 ± 2.5 mm among patients without direct-triggered episodes. The number of teeth with mobility was 37 (24.2%).

The correlation between occurrence of acute phase of chronic periodontitis without direct-triggered episodes and meteorological parameters is shown in Table 2. Based on the correlation of the results, the final model was predicted using the ARIMA model (Table 3). The independent covariants, namely maximum hourly range (decrease) of barometric pressure, maximum hourly range (increase) of temperature, and maximum daily wind speed were significantly associated with occurrence of acute phase of chronic periodontitis without direct-triggered episodes (*p* < 0.05). The determination coefficient (*r*^2^) of the final model was 0.031% or 3.1% of the variations in occurrence of acute phase of chronic periodontitis without direct-triggered episodes over the study period were explained by the factors included in the model.

**Table 2:** Significant cross correlations with acute phase of chronic periodontitis without direct-triggered episodes.

Parameter	*r*	*P* Value
**Wind speed (m/s)**		
Mean daily wind speed	0.062	<0.001
Maximum daily wind speed	−0.015	0.027
Minimum daily wind speed	Omitted	
**Barometric pressure (hPa)**		
Mean daily barometric pressure	Omitted	
Maximum daily barometric pressure	Omitted	
Minimum daily barometric pressure	Omitted	
Daily range of barometric pressure	0.019	<0.001
Maximum hourly increase in barometric pressure	0.133	0.002
Maximum hourly decrease in barometric pressure	0.099	<0.001
Total hours of bright daily sunshine (h)	Omitted	
Total daily rainfall (mm)	Omitted	
**Temperature (°C)**		
Mean daily temperature	−0.005	0.015
Maximum daily temperature	−0.005	0.009
Minimum daily temperature	−0.004	0.015
Daily range of temperature	Omitted	
Maximum hourly increase in temperature	−0.05	0.018
Maximum hourly decrease in temperature	Omitted	
**Humidity (%)**		
Mean daily humidity	Omitted	
Maximum daily humidity	Omitted	
Minimum daily humidity	Omitted	
Daily humidity range	Omitted	

**Table 3:** Multivariate time-series analysis model for acute phase of chronic periodontitis without direct-triggered episodes (*r*^2^ = 0.031).

Term	Lag Time ^a^	Coefficient (S.E.) ^b^	T Ratio	*P* Value
Maximum hourly decrease in barometric pressure	2	0.098 (0.031)	3.156	0.002
Maximum hourly increase in temperature	1	0.046 (0.018)	2.473	0.014
Maximum daily wind speed	3	−0.015 (0.006)	−2.501	0.013

^a^ Represents the delay necessary to observe the effect (in days).^b^ Indicates the size and the direction of the effect.

## 4. Discussion

To the best of our knowledge, this is the first epidemiological study to assess the relationship between occurrence of acute phase of chronic periodontitis and meteorological variables during the maintenance phase of periodontal treatment. In this study, ARIMA model analysis showed that maximum hourly decrease in barometric pressure, maximum hourly increase in temperature, and maximum daily wind speed were significantly associated with occurrence of acute phase of chronic periodontitis. In this model, 3.1% of the variations in occurrence of acute phase of chronic periodontitis over the study period were explained by these factors. Our results provide new findings in the acute phase of chronic periodontitis development. In addition, occurrence of acute phase of chronic periodontitis seems to occur one to three days after changes in meteorological variables. It is feasible that there is a response time lag in which meteorological variables induce pathological changes in periodontal tissue. In the occurrence of acute phase of chronic periodontitis, the first step may be the invasion of bacteria into the soft tissues surrounding the periodontal pocket, which will develop an inflammatory process [34]. Bacterial pathogens attract inflammatory cells to induce chemokines and cytokines, and modulate the inflammatory response during 2–72 h [35,36,37,38]. These findings might support the time lag.

In this study, maximum hourly decrease in barometric pressure was related to occurrence of acute phase of chronic periodontitis. A case report suggested that sudden decreases in barometric pressure when on an airplane can influence the disease activity of patients with acute apical periodontitis [39], which supports our findings. Barometric pressure may have an effect on myopia [40], passenger discomfort on aircrafts [41], sleep disordered breathing [42], deep venous thrombosis [43], and oral pain [28,44]. In an animal model, decreases in barometric pressure induce pain and increase blood pressure and heart rate, suggesting the direct effects of barometric pressure on sympathetic nerve activity and the indirect effects of activation of the peripheral nociceptive and/or mechano-receptive fibers [45]. Although it is difficult to explain the relationship between barometric pressure and occurrence of acute phase of chronic periodontitis, there may be a possible mechanism or hormonal changes [46]. Decreases in barometric pressure possibly modulate the environment of hormones such as adrenaline [46]. Some hormones have direct effects on the growth of periodontitis-related bacteria in vitro [47]. Thus, decreases in barometric pressure might indirectly contribute to occurrence of acute phase of chronic periodontitis. However, further studies are required.

Changes in temperatures contribute to the observed temperature-related mortality [48,49,50,51] and increased cardiovascular and respiratory risk [52,53]. Occurrence of acute phase of chronic periodontitis was also related to maximum hourly increase in temperature in this study. Changes in temperature can control blood biomarkers. For example, cumulative increases in fibrinogen and plasminogen activator inhibitor type 1 in diabetes patients are observed in association with a 5 °C temperature decrement [54]. It is also known that increased C-reactive protein and interleukin-6 occur with a 10 °C temperature decrement [55]. Therefore, the inflammatory process in periodontal tissue may be partially affected by the temperature-related changes in blood biomarkers. Further studies are required to explore the exact mechanisms that promote the association between occurrence of acute phase of chronic periodontitis and changes in temperature.

Wind speed may have an indirect effect on the severity and frequency of air pollution occurrence in respiratory allergic disease [56]. It is also reported that higher wind speed provides a small increase in the risk of back pain [57]. In the present study, occurrence of acute phase of chronic periodontitis was negatively related to maximum daily wind speed. This supports the concept that not only barometric pressure and temperature but also wind speed can affect health, including periodontal condition.

The present comprehensive epidemiological study describes the influence of changes in meteorological valuables on occurrence of acute phase of chronic periodontitis and yields evidence that this can be a phenomenon of everyday life. It is commonly accepted that the weather itself may influence the well-being of individuals. Therefore, dentists should be aware of the relationship and inform their patients about the weather being one of the probable reasons for certain complaints.

In this study, the acute phase of chronic periodontitis during supportive periodontal therapy was 1.84% of all patients. Previous studies report that the prevalence of periodontal abscess is 1.04%–27.5% [34,58,59,60,61,62]. The prevalence in this study was within the range, which suggests that participants were not limited to a specific group and the results may be generalized to a various populations.

Our study has some limitations. First, the experimental period (two years) may be too short to investigate the seasonal effects. A long-term study will be required to clarify it. Second, the number of patients was small in this study. Future study needs a large number of patients. Third, meteorological data were only acquired from one area. Because other areas where occurrence occurred were quite few, we selected cases in a limited area. A multicenter study including more areas will be required. Fourth, 3.1% of the variations in occurrence of acute phase of chronic periodontitis without direct-triggered episodes were explained by meteorological factors. However, since 41.4% of occurrence was of unknown origin in this study, other explanation such as a minor shift in the microorganisms should be considered. Further studies are needed to clarify the details of acute phase of chronic periodontitis without direct-triggered episodes.

## 5. Conclusions

Maximum hourly decrease in barometric pressure, maximum hourly increase in temperature, and maximum daily wind speed were significantly associated with occurrence of acute phase of chronic periodontitis without direct-triggered episodes. In the model, 3.1% of the variations in the occurrence of acute phase of chronic periodontitis over the study period could be explained by these factors.
